# Breast ironing in Africa: a scoping review protocol

**DOI:** 10.3389/frph.2026.1791767

**Published:** 2026-04-02

**Authors:** Ephraim Senkyire, Robert Kogi, Gloria Senkyire, Jude Ameyaw, Magdalena Ohaja, Emmanuel Lamptey, Ernestina Asiedua

**Affiliations:** 1 Department of Nursing and Midwifery, Ga West Municipal Hospital-Ghana Health Service, Accra, Ghana; 2 Department of Public Health, Ghana Health Service, District Health Directorate, Kukuom, Ahafo, Ghana; 3 Department of Accountancy, Faculty of Business and Management Studies, Sunyani Technical University, Sunyani, Ghana; 4 Department of Nursing and Midwifery, Elmina Polyclinic, Ghana Health Service, Cape Coast, Ghana; 5 School of Nursing and Midwifery, University of Galway, Galway, Ireland; 6 School of Basic and Biomedical Sciences, University of Health and Allied Sciences, Ho, Ghana; 7 School of Nursing and Midwifery, University of Ghana, Accra, Ghana

**Keywords:** adolescent girls, Africa, breast flattening, breast ironing, breast massaging, gender-based violence, harmful traditional practices, scoping review

## Abstract

**Background:**

Breast ironing is a harmful cultural practice reported in some African communities and, although often culturally justified, it is increasingly recognised as a violation of the rights of affected girls. This review aims to answer: (1) What is the prevalence of breast ironing? (2) What motivations are reported? (3) What are its health and social impacts?

**Objective:**

The objective of this scoping review is to map evidence on prevalence, drivers, consequences, and interventions related to breast ironing.

**Method and analysis:**

Searches will be conducted in MEDLINE (PubMed), CINAHL (EBSCOhost), Scopus, and Web of Science, with grey literature identified through Google Scholar and institutional repositories, including African Journals Online (AJOL). The review will follow the Joanna Briggs Institute (JBI) scoping review methodology, and study selection will be reported in accordance with PRISMA-ScR guidelines. Two reviewers will independently screen titles/abstracts and full texts, while two reviewers will extract data. Findings will be synthesised using a descriptive analytical approach and presented as a structured narrative summary.

**Discussion:**

This review will strengthen the existing evidence base on the impact of breast ironing across Africa. Across studies, common motivations are expected to centre on perceived protection from sexual attention, early pregnancy, sexual violence, or forced marriage, while consistently reporting substantial physical harms—such as pain and tissue damage—and psychological consequences, including fear, shame, trauma, and loss of bodily autonomy. Recognising the practice within gender-based violence legislation may enhance legal accountability and protection for girls, while targeted training for healthcare providers and community-based education initiatives may support efforts to prevent the practice and mitigate its health and social consequences.

## 1 Introduction

Breast ironing (BI) is the practice of pressing, hammering, or massaging the developing breasts of adolescent girls between the ages of 10 and 19 years with hot tools such as pestles, spatulas, or stones (1). This practice is mostly reported in Cameroon, but also found in countries like Nigeria, Togo, Guinea-Bissau, and some other Central and West African countries (2). However, the practice may be underreported in these regions due to limited research. This practice is normally carried out by mothers or female guardians with the intention of protecting the victims (girls) from unwanted pregnancies, forced marriages, unwanted sexual attention and sexual assaults (3) yet the consequence is harmful and violates human rights. Although the rationale is perceived as protective by perpetrators or culturally motivated, breast ironing constitutes a gendered form of violence (4, 5), resulting in serious health risks such as burns, physical disfigurement, pain, infection, breast tissue trauma, breast deformities, violation of bodily autonomy and psychological trauma such as anxiety, depression, post-traumatic stress (6–8).

Approximately, BI affects 3.8 million women globally, with an estimated 25%–50% of girls in Cameroon (8). Evidence also suggested that BI is performed in the diaspora, particularly among African immigrant populations in countries like the UK (5, 9), with over 1000 girls being at risk (8). Few countries, such as Cameroon, Côte d'Ivoire and Nigeria, have passed specific laws outlawing the practice or set aside funds for the prevention of BI, despite the UNFPA identifying it as one of five underreported forms of GBV in Africa (10). This practice remains less recognised in academic discourse and public health policy due to a lack of knowledge, cultural normalisation, and policy inertia (6, 7, 10). Also, previous studies have limited the independent evaluation of breast ironing by frequently grouping it with other practices, including female genital mutilation (3, 11, 12). The combined effect of BI with more general systemic problems like gender discrimination, poverty, and low educational achievement among those affected may result in long-term cycles of disadvantages (13). The need for a scoping review is to map limited peer-reviewed prevalence, fragmented evidence, identify research gaps, and inform future primary studies or interventions. Hence, the objective of this review is to map evidence on prevalence, drivers, consequences, and interventions related to breast ironing and answer the following questions: What is the reported prevalence of breast ironing across African countries? What are the motivations and drivers reported by perpetrators and communities? What are the physical, psychological, and social consequences for affected girls? And what interventions have been implemented to prevent or address the practice?

## 2 Materials and methods

### 2.1 Population

The review will focus on adolescent girls, their mothers/female guardians, healthcare providers or community workers with experience related to the practice.

### 2.2 Study design

This scoping review will be conducted in accordance with the Joanna Briggs Institute (JBI) methodology for scoping reviews (14). The JBI framework provides a rigorous and transparent approach to systematically identifying, mapping, and synthesising evidence across diverse study types, particularly in underexplored areas like breast ironing. We will conduct the analysis using a qualitative content analysis (15) and descriptive statistics (16) and report using the Preferred Reporting Items for Systematic Reviews and Meta-Analyses Extension for scoping reviews (PRISMA-ScR) (17). The study is registered at Open Science Framework (https://doi.org/10.17605/OSF.IO/GY4FW)

### 2.3 Search strategy

An experienced librarian reviewed the search strategy to ensure its accuracy and completeness. Search optimisation will involve combining Medical Subject Headings (MeSH) with keywords such as “breast ironing,” “motivation,” “practice,” “Africa,” and their various iterations. The Boolean operators ‘AND’ and ‘OR’ will be incorporated alongside these MeSH/MH and keyword terms to broaden the search scope. The search parameters will be adjusted to include only recent articles published from 2012 to 2025, published in English, and with abstracts available. The search strategy will be adapted to each database's syntax and subject headings, and for a grey literature search. See Table 1.

**Table 1:** CINALH search strategy.

**Concept 1: Practice/Motivations/Social Factors** **Subject Headings:** "Health Behaviour"[MH] OR "Social Values"[MH] OR "Cultural Characteristics"[MH] OR "Motivation"[MH] **Keywords:** Practice* OR perform* OR influence* OR contribut* OR facilitat* OR enable* OR reason* OR motivation* OR "cultural belief*" OR "social factor*"Concept 2: Breast Ironing
**Concept 2: Breast Ironing** **Subject Headings:** No specific MH term available **Keywords:** "Breast ironing" OR "breast flattening" OR "breast massaging" OR "breast pounding" OR "breast pressing" OR "breast rubbing" OR "chest ironing" OR "breast mutilation"
**Concept 3: Location (Africa)** **Subject Headings:** "Africa"[MH] OR "Africa South of the Sahara"[MH] **Keywords:** Africa OR "West* Africa*" OR Benin OR "Burkina Faso" OR "Cape Verde" OR "Côte d'Ivoire" OR Gambia OR Ghana OR Guinea OR "Guinea-Bissau" OR Liberia OR Mali OR Mauritania OR Niger OR Nigeria OR "Saint Helena" OR Senegal OR "Sierra Leone" OR Togo OR "Central Africa*" OR Angola OR Cameroon OR "Central African Republic" OR Chad OR Congo OR "Democratic Republic of Congo" OR "Equatorial Guinea" OR Gabon OR "São Tomé and Príncipe" OR "Southern Africa*" OR Botswana OR Eswatini OR Lesotho OR Madagascar OR Malawi OR Mauritius OR Mozambique OR Namibia OR "South Africa" OR Zambia OR Zimbabwe OR "Northern Africa*" OR Algeria OR Egypt OR Libya OR Morocco OR Sudan OR Tunisia OR "Western Sahara" OR "Eastern Africa*" OR Burundi OR Comoros OR Djibouti OR Eritrea OR Ethiopia OR Kenya OR Rwanda OR Seychelles OR Somalia OR "South Sudan" OR Tanzania OR Uganda OR "Sub-Sahara Africa"

### 2.4 Inclusion and exclusion criteria

This review will include data sources published in English between 2012 and 2025, to capture the most recent evidence focusing specifically on African countries. This period marks a significant increase in the documentation of gender-based violence and harmful traditional practices, coinciding with intensified global advocacy for women's and girls’ rights (6). The review will consider observational studies, legal commentary, health reports, grey literature, viewpoints, systematic reviews, narrative reviews, and qualitative interviews. Studies conducted outside the African context will be excluded, and studies that do not address the defined concept and population of the practice. The PCC framework (18) will be used to guide the selection of relevant literature. See Table 2.

**Table 2:** PCC framework.

PCC	Inclusion	Exclusion
Population	Adolescent girls (10−19 years) who have undergone breast ironing, their mothers/female guardians, healthcare providers or community workers with experience related to the practice. A retrospective account from adult women who experience BI.	Studies involving participants outside the defined population, including adult women (≥20 years) who did not experience breast ironing, male adolescents, or individuals with no direct or indirect experience of the practice.
Concept	Studies that mention breast ironing only in passing, without substantive data on the impact of the practice	Studies that do not mention breast ironing
Context	Studies conducted in African countries	Studies conducted outside Africa

### 2.5 Information sources

These will include searches of MEDLINE (PubMed), CINAHL (EBSCOhost), Scopus, and Web of Science. Searches will also be conducted in Google Scholar and institutional repositories such as UN Women and African Journals Online (AJOL) to capture grey literature using a combination of the defined key terms and limiters. The first 200 results will be screened, consistent with recommended systematic review practice (19). Titles and snippets will be screened for relevance. Although relevant studies will be missed, the date ranges, keywords, or the advanced search tool will be used to ensure that relevant literature is not missed. Where potentially eligible, full texts will be retrieved, and duplicate records will be removed before screening. To ensure comprehensiveness, the reference lists of all included sources of evidence will be screened for additional relevant studies. Furthermore, the reference lists of systematic reviews and reports on related topics (e.g., harmful traditional practices, gender-based violence, adolescent health in Africa) will also be examined.

### 2.6 Study selection

All identified citations will be collated and uploaded to Zetoro Reference Manager (20), then to the Covidence software (21). The study selection process will be reported using the PRISMA flow chart (22). Two reviewers will independently assess the titles, abstracts, and full texts of selected articles against the inclusion criteria using Covidence software. A third reviewer will resolve any disagreements between the reviewers at each stage of the selection process.

### 2.7 Data extraction

Data will be extracted from the included papers by two independent reviewers using a predefined data extraction template developed by the reviewers. Data will be extracted on the following: author's name, year, country (specific location), aim, study design, sample size, key findings related to PCC, and limitations. See Table 3. Disagreements between the reviewers will be resolved through discussion with a third reviewer.

**Table 3:** Data extraction form.

Author, year	Country/specific location	Focus/Aim	Design/ Methodology	Sample size	Key findings	Limitations
						

## 3 Outcome

The review will synthesise evidence on the experiences of victims and their families, the health effects, and the human rights implications of breast ironing.

## 4 Data analysis

The descriptive-analytical approach (23) will be used in this study. This method describes, summarises, and interprets data to explain what a phenomenon entails, often using qualitative content analysis or quantitative summary statistics. It seeks to provide a clear and comprehensive account of events or experiences (23). Thus, qualitative content analysis (15) will be used to analyse qualitative data inductively, and quantitative data will be summarised descriptively using frequencies (16). The findings will be integrated across studies by using a joint display (24).

## 5 Results

The review will present a narrative synthesis of the findings from the selected studies, structured around the review questions. Various studies have acknowledged the experiences of victims and parents of breast ironing, the effects and human rights abuses of these victims, which will be thoroughly reported. Thus, it will help policymakers and stakeholders to outline particular policies on reproductive and sexual health of adolescent girls to promote health for all and ensure human rights are upheld. The selection of eligible studies will be illustrated using the PRISMA-ScR flow diagram, and tables of study characteristics will depict vital attributes of individual included studies.

## 6 Discussion

The practice of breast ironing should be reinterpreted not merely as a cultural form of protection, but as a structural expression of gender inequality and gender-based violence against women and girls. Framing the practice within this broader socio-structural context allows for a more critical understanding of how patriarchal norms, control of female sexuality, and community-level anxieties about early pregnancy shape its persistence across settings. Building on previous reports/research, this review moves beyond descriptive accounts to provide a focused, continent-wide synthesis dedicated exclusively to breast ironing. Based on preliminary literature, this review anticipates identifying highly variable prevalence estimates across countries and regions, reflecting differences in study design, sampling approaches, and sociocultural contexts. It is also expected that rigorous, population-based prevalence data will be limited, with much of the existing evidence derived from small-scale qualitative studies, NGO reports, or media documentation.

Across studies, consistent themes are likely to emerge regarding motivations for the practice—particularly the perceived need to protect girls from sexual attention, early pregnancy, sexual violence, or forced marriage. Simultaneously, the review expects to find recurring reports of significant physical harms (such as pain, tissue damage, and long-term breast complications) and psychological harms, including fear, shame, trauma, and diminished bodily autonomy. Furthermore, it is anticipated that there will be few documented or formally evaluated interventions, highlighting a substantial gap in evidence on prevention and response strategies.

This review will advance knowledge by providing the first comprehensive mapping of available evidence on breast ironing across Africa, systematically consolidating findings from diverse study designs and sources. Thus, it will identify critical gaps for future primary research, including the need for robust prevalence studies in under-documented countries and rigorous evaluations of community-based or policy-level interventions. By synthesising evidence on the health, psychosocial, and human rights implications of the practice, the review will also inform policy dialogue and advocacy efforts, supporting rights-based, context-sensitive strategies aimed at prevention, protection, and structural change.

Significantly, the findings have significant legal and policy implications. In countries where the practice persists, its inclusion in GBV legislation could strengthen legal accountability and protection mechanisms for girls. Explicit classification within child protection and violence prevention laws will also support enforcement, improve reporting systems, and facilitate allocation of resources for prevention and survivor support. Furthermore, integrating breast ironing into national reproductive health, adolescent health, and safeguarding policies will ensure that it is no longer treated as a trivial or entirely cultural issue. Regional bodies such as the African Union can also incorporate the issue into broader gender equality and child protection agendas to promote consistent policy responses across member states.

Healthcare providers, including nurses and midwives, are strategically positioned to detect early signs during routine consultations. Strengthening their capacity through pre-service and in-service training (trauma-informed and culturally sensitive care, confidential reporting and referral pathways, recognising signs and complications, and integrating screening into adolescent and reproductive health services) will improve early identification, documentation, and survivor-centred care.

Community interventions should move beyond criticism to culturally informed engagement that addresses underlying fears about sexuality and social stigma. Empowering girls through education and safe reporting mechanisms is equally critical. Future research will focus on documenting prevalence data across all African regions, including men and community perspectives, reporting policy enforcement outcomes and conducting intervention-based or longitudinal studies.

## 7 Strengths and limitations

The review will benefit from a comprehensive search, adherence to the JBI framework and PRISMA-ScR for reporting selected studies. However, the review will be limited to English-language published studies, a possible limitation of non-indexed/local literature, exclusion of relevant French- and Portuguese-language literature, particularly from West and Central African countries where such languages are widely used. In addition, the grey literature search limit may lead to the omission of pertinent reports, policy documents, or local evidence. There is also a potential risk of publication bias, as studies documenting the existence or harms of the practice may be more likely to be published than those reporting its absence or lower prevalence. Furthermore, the anticipated heterogeneity of study designs, methodologies, and outcome measures may limit the depth of synthesis. Although no formal quality appraisal is planned, the inclusion of studies without critical appraisal may affect the interpretive strength of the findings.
